# Evaluation of *Quillaja brasiliensis* Saponin-Based Nanoparticles Combined with Leucine Aminopeptidases for Immunoprotection of Sheep Against *Fasciola hepatica*

**DOI:** 10.3390/vaccines13101008

**Published:** 2025-09-26

**Authors:** Jackeline Checa, Antonella Goyeche, Renzo Vettorazzi, Pablo Alonzo, Oscar Correa, Walter Norbis, Estela Castillo, Martin Cancela, Andrea Rossi, Fernando Silveira, Gabriela Maggioli

**Affiliations:** 1 Laboratorio de Biología Parasitaria, Instituto de Higiene-Facultad de Ciencias, Universidad de la República, Av. Dr Alfredo Navarro, Montevideo 11600, Uruguay; 2 Campo Experimental, Instituto de Higiene, Universidad de la República, Canelones 46500, Uruguay; 3 Parasitología Veterinaria, Facultad de Veterinaria, Universidad de la República, Ruta 8, km 18, Montevideo 11600, Uruguay; 4 Instituto de Biología, Departamento de Biología Animal (FREP), Facultad de Ciencias, Universidad de la República, Iguá 4225, Montevideo 11400, Uruguay; 5 Laboratório de Genômica Estrutural e Funcional, Centro de Biotecnologia, Universidade Federal do Rio Grande do Sul, Porto Alegre 91501-970, Brazil; 6 Unidad Académica de Desarrollo Biotecnológico, Instituto de Higiene, Facultad de Medicina, Universidad de la República, Av. Dr Alfredo Navarro 3051, Montevideo 11600, Uruguay

**Keywords:** adjuvants, fasciolosis, leucine aminopeptidases, protective immune response, ruminants, vaccines

## Abstract

**Background:** *Fasciola hepatica* causes important economic losses in ruminants with only pharmacological treatments currently available, which produces several secondary problems. Because of this, vaccines have become an interesting alternative. Leucine aminopeptidases (LAPs) are attractive vaccine targets against fasciolosis since they play essential roles in the parasite such as host invasion and nutrient acquisition. To characterize immune responses, we produced two recombinant *F. hepatica* LAPs (*Fh*LAP1 and *Fh*LAP2), formulated with ISCOM-matrices (IMXs) nanoparticles from *Quillaja brasiliensis* saponins. **Methods:** Forty female Corriedale sheep were assigned to four groups (*n* = 10): *Fh*LAP1/IMX, *Fh*LAP1/*Fh*LAP2/IMX, IMX (control), and *Fh*LAP1/Adj50 (Adjuvac 50). Animals received two subcutaneous immunizations at weeks 0 and 4 and were challenged orally with 200 metacercariae at week 6. **Results:** *Fh*LAP1 and *Fh*LAP1/*Fh*LAP2 induced specific IgG responses, with the predominance of the IgG1 response. However, these responses were lower than those generated by *Fh*LAP1 formulated with Adj50. A qPCR analysis revealed that *Fh*LAP1/IMX stimulated a Th1-type response profile before the challenge, but this profile was not sustained after infection. The post-infection profile of *Fh*LAP1/*Fh*LAP2/IMX was more congruent with expected values despite not achieving a robust IFN-γ expression. No significant differences in the fluke burden were observed. **Conclusions:** Further research on the optimal antigen/adjuvant combination in ruminants is encouraged. For instance, a higher concentration of adjuvant in the formulation used in this work may enhance the strength and duration of the inflammatory response and improve protective immunity against fasciolosis.

## 1. Introduction

Fasciolosis, an infection caused by *Fasciola hepatica*, leads to significant economic losses in livestock [1]. This is due to the increased susceptibility to secondary infections, reduced meat, wool, and milk production, interference with fertility, and expenses related to the application of flukicide treatment [1,2]. Furthermore, human fasciolosis is considered a re-emerging disease [3].

Controlling *F. hepatica* infection relies on treatment with triclabendazole (TCBZ) [4]. However, the high cost, reported cases of drug resistance, the presence of toxic residues in food, and, additionally, the impact they may have on the local environment, create significant obstacles for the marketing of these products [5,6,7]. Vaccination emerges as an alternative capable of providing greater long-term protection and sustainability, as it does not generate toxic residues for consumption and is also not detrimental to the environment [8]. However, to develop new vaccines against *F. hepatica*, it is crucial to enhance and deepen our understanding of the protective immune response mechanisms in ruminants [8].

During the early stages of fasciolosis, immune response is generally characterized as a mixed Th1/Th2 response, marked by an increase in certain cytokines such as IFNγ, IL4, IL10, and TGFβ in the host. As the infection advances, the Th2 response is amplified, accompanied by the suppression of Th1, which allows for a prolonged infection potentially dependent on IL4 [9].

Helminth peptidase enzymes are crucial factors for parasite establishment within their hosts. Leucine aminopeptidases (LAPs), belonging to the Zn-metalloproteases of the M17 family, have been reported to be involved in tissue invasion and parasite nutrition [10,11,12,13,14,15,16]. For this reason, LAPs have been proposed as potential vaccination antigens to control helminth infections. Previous studies demonstrated the protective potential of the recombinant leucine aminopeptidase 1 (*Fh*LAP1) against fasciolosis in male sheep (74–86%) [17]. Moreover, a second *F. hepatica* leucine aminopeptidase (*Fh*LAP2), expressed in earlier stages of the parasite [18,19], showed a 70% protection against *F. hepatica* infection in a murine model [20].

Another essential component in vaccine development is adjuvant selection, primarily aiming to enhance potency by activating innate immunity and promoting controlled inflammation [21]. Nanoparticle adjuvants offer advantages over traditional formulations by inducing rapid and long-lasting cellular and humoral immunity, enabling multiple routes of administration, providing thermal stability at room temperature, and preserving antigen functionality. These features reduce storage costs, facilitate global distribution, and highlight their potential to improve vaccine efficacy in agricultural species [22]. Recently, nanoparticle-based formulations containing ferritin, chitosan, or poly(lactic-co-glycolic) acid (PLGA) showed the induction of a strong immune response that could protect against *Echinococcus granulosus*, *Schistosoma japonicum*, and *Haemonchus concortus* infection [23,24,25]. Other studies report ISCOMs, nanoparticles formed by Quil A saponin extracted from the bark of *Quillaja saponaria*. These nanoparticles can encapsulate antigens or be delivered as ISCOMATRIXs, and their particulate and hydrophobic nature enhance the efficient antigen uptake by APCs [26]. Our group had previously reported that the saponin fraction (QB) extracted from the leaves of *Quillaja brasiliensis,* a plant native to Uruguay, can stimulate strong immune responses when formulated with viral antigens [21]. This fraction possesses effective adjuvant properties that make it competitive with market products. To reduce the inherent toxic effects of QB, it is included in micellar formulations called ISCOM-matrices (immunostimulatory complexes) (IMXs). This results in a formulation of nanoparticles which has been reported to successfully work as an early immune response activator, triggering both systemic and mucosal responses [27,28]. More importantly, tests in mice showed that IMX formulations promote high levels of IgG1/IgG2a/IgG2b antibody titers and a Th1-type response (IL2 and INFγ), which is the required immune phenotype for effective control against fasciolosis [21].

Vaccination studies in ruminant livestock species such as cattle, sheep, and goats showed a Th1-type protective response along with high levels of IgG2 [29]. Protection has also been observed, associated with high levels of IgG2 and IgG1 antibodies, indicating a mixed Th1/Th2-type response [17,30,31]. Although no data on the cytokine profile generated in a protective response has been published, it has been suggested that an effective vaccine against fasciolosis should reduce the regulatory effects induced by the parasite and generate a Th1-type or a mixed Th1/Th2-type response throughout the *F. hepatica* infection. This has been explored in previous studies on vaccination against fasciolosis in ruminants, facilitating advances in understanding the mechanisms of immune protection. For example, the recombinant *Fh*LAP1 produced in our laboratory, combined with different commercial adjuvants, has shown relevant protection results in male sheep (74–86%) [17]. Other antigens with encouraging results include CL1/CL2 mimotopes (47%) and the CL1 mimotope (51%) [31,32]. However, the persistent problem is that none of these promising results have been reproducible in female sheep or cattle, nor have they been validated in field trials [33,34].

In this study, we report the results of a vaccination trial conducted in female sheep using a novel formulation including two leucine aminopeptidases, *Fh*LAP1 and *Fh*LAP2, the latter being expressed in early intra-mammalian developmental stages of the parasite. The antigens were formulated with IMX nanoparticles derived from *Q. brasiliensis* saponins, alongside another oil-based adjuvant.

## 2. Materials and Methods

### 2.1. Production of Recombinant Proteins

Recombinant expressions of the *Fh*LAP1 (AY644459) and *Fh*LAP2 (ON428200) were performed according to Checa et al. [20] and Maggioli et al. [16,17], with modifications. For *Fh*LAP1, we prepared a new expression plasmid pET-*Fh*LAP1 inserting the *Fh*LAP1 gene into the *BamH*I/*Sal*I site of pET28a vector (Novagene). For *Fh*LAP2, we constructed a new expression plasmid pET-*Fh*LAP2 inserting the *Fh*LAP2 fragment into the *Sac*I/*Sal*I site of pET24a vector (Novagene).

For both *Fh*LAP1 and *Fh*LAP2 recombinant expressions, we used the same protocol. Briefly, *E. coli* strain BL21 (DE3) cells were transformed with the respective plasmids, and the recombinant clone was grown in LB media with kanamycin (50 μg/mL) to an optical density (OD) 600 nm of 0.8, and recombinant protein production was induced with 0.4 mM IPTG for 24 h at 25 °C.

For *Fh*LAP1 and *Fh*LAP2 protein purification, we used two different protocols, as described in Checa et al. [20] and Maggioli et al. [16,17]. For *Fh*LAP1, the bacterial culture was centrifuged, the pellet resuspended in 50 mM Tris-HCl pH 8.5, 100 mM NaCl, and 5 mM imidazole, containing 1 mg/mL lysozyme, and sonicated (10 pulses of 1 min with 1 min pauses). The lysates were centrifuged for 30 min at 20,000× *g*, and supernatants were applied to a nickel–nitrilotriacetic acid column (GE Healthcare, Uppsala, Sweden), washed with 50 mM Tris-HC pH 8.5, 100 mM NaCl, and 5 mM imidazole and eluted in imidazole at 20, 50, 200, and 400 mM.

To obtain recombinant *Fh*LAP2, the bacterial culture was centrifuged, and the protein was recovered from the inclusion bodies. Pellet was resuspended in 50 mM Tris pH 8.5, 100 mM NaCl, and 1.5% N-Lauroylsarcosine sodium salt, shaken gently for 1 h, sonicated and centrifuged for 30 min at 20,000× *g*, and 2% Triton X-100 (AppliChem, Darmstadt, Germany) was added to the supernatant and was incubated within nickel–nitrilotriacetic acid column (GEHealthcare) for 1 h. Then, it was washed with 50 mM Tris pH 8.5, 100 mM NaCl, and 5 mM Imidazole and eluted in imidazole at 20, 50, and 200 mM.

The fractions containing both recombinant proteins were dialyzed against PBS and stored at 4 °C. The protein concentration was determined using the bicinchoninic acid assay (BCA1 Kit, Sigma-Aldrich, St. Louis, MO, USA). The purity of recombinant enzymes was examined using Coomassie-stained 10% SDS-PAGE gels, under reducing conditions. The identity of each recombinant protein was confirmed by MALDI-TOF MS/MS at the Proteomic Unit of the Pasteur Institute of Montevideo.

### 2.2. ISCOM-Matrices Adjuvant: Preparation and Characterization

QB (A. St.-Hil. et Tul) Mart. leaves were collected in Parque Battle, Montevideo, Uruguay (−34.89302, −56.15727) (voucher MVFQ 4321, deposited at the Herbarium of the Facultad de Química, Universidad de la República, Montevideo Uruguay y). Extraction and purification of saponin fractions were carried out as previously described [35]. IMX was prepared using the dialysis method previously described [36], and nanoparticles obtained were sterilized by filtration using a 0.22 µm syringe filter and maintained at 4 °C.

The preparation was visualized by transmission electron microscopy (TEM) with a JEMM-2100 (JEOL Ltd., Tokyo, Japan) high-resolution transmission electron microscope using a previously described methodology [36].

### 2.3. Immunoprotection Trial

Forty female Corriedale sheep (eight months old) were housed in the fluke-free experimental field (Campo experimental del Instituto de Higiene, Canelones, Uruguay. Animals were isolated in 4 groups of *n* = 10 with food and water *ad libitum*. Animal manipulation was performed in accordance with “Comisión Honoraria de Experimentación Animal” (CHEA) guidelines and was approved by the Uruguayan University Research Ethics Committee (approval number 1046; 22 May 2020).

**Figure 1:**
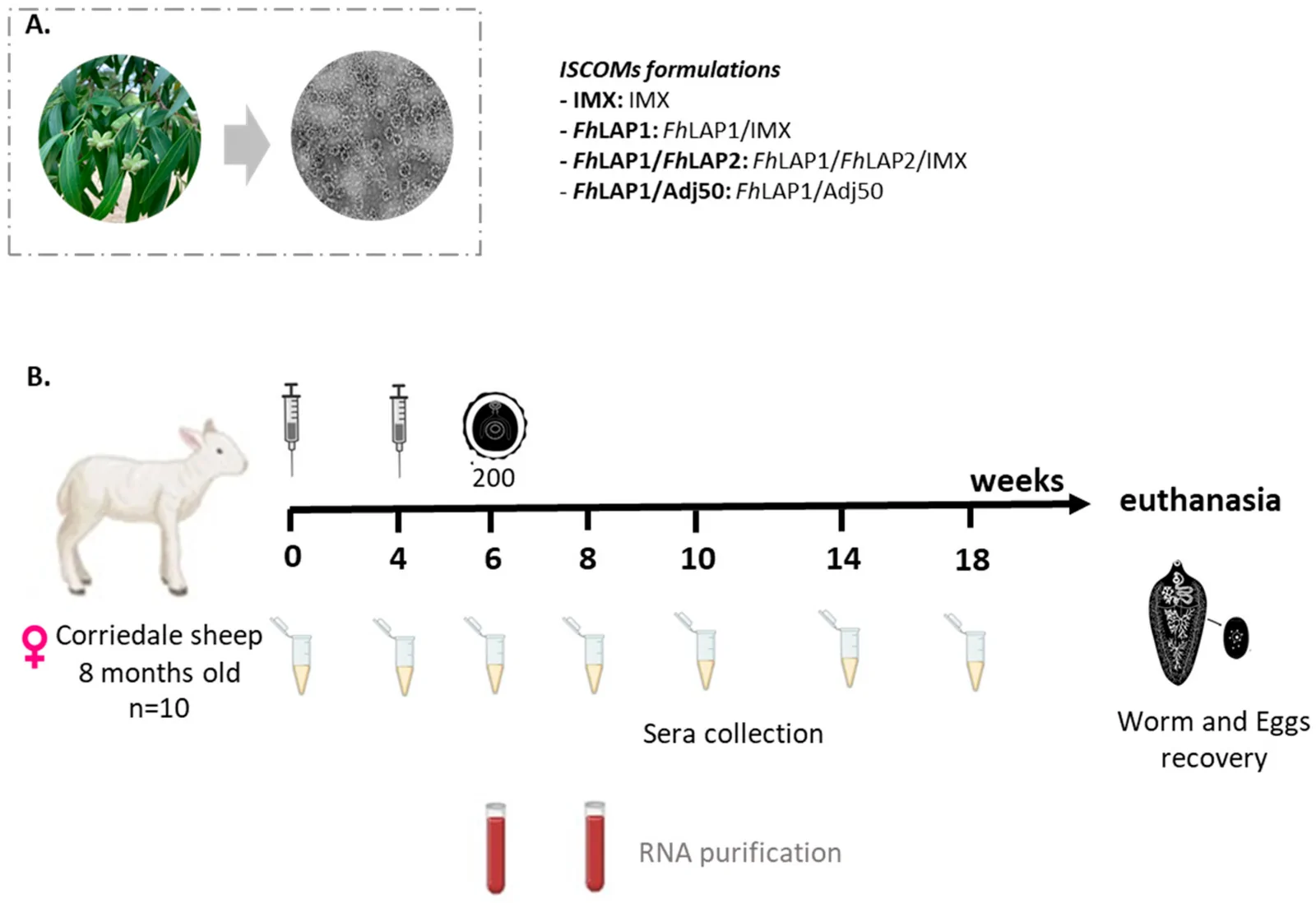
(**A**) IMX: nanoparticles (40 nm) formulated with *Q. brasilliensis* (QB) purified saponin fraction (without antigen) by the dialysis method and visualized by HR-TEM. Negative staining preparation was visualized by TEM with Joel JEM 2100, LaB6-HRSTEM. (**B**) Scheme of the vaccination and challenge experiments. Female Corriedale sheep were immunized s.c on weeks 0 and 4 with *Fh*LAP1/IMX; *Fh*LAP1/*Fh*LAP2/IMX; IMX; or *Fh*LAP1/Adj50. Two weeks later, the first three groups were orally challenged with 200 metacercariae of *F. hepatica*. The blood samples were collected every two weeks. At week 20, animals were euthanized.

The vaccinated groups were received 2 subcutaneous doses of 100 µg of both purified recombinant *Fh*LAPs formulated with 50 µg IMX (at weeks 0 and 4) as follows: group *Fh*LAP1 received *Fh*LAP1/IMX; group *Fh*LAP1/*Fh*LAP2 received *Fh*LAP1/*Fh*LAP2/IMX; and control group IMX received IMX alone. Additionally, we included group *Fh*LAP1/Adj50, which received 100 µg of *Fh*LAP1 adjvanted with Adjuvac 50 (Laboratorio VIRBAC Uruguay), with which we had previously achieved a high level of protection in male sheep (Figure 1). At week 6, sheep from the groups *Fh*LAP1, *Fh*LAP1/*Fh*LAP2, and IMX were orally challenged with 200 metacercariae. Blood was collected from all animals before the first immunization and then biweekly until the time of euthanasia. The serum was obtained and stored at −80 °C until the ELISA analysis. At 14 weeks after infection, animals were euthanized. The livers were recovered, and lesions were scored based on the criteria described by Ramamoorthi et al. [37], with modifications: Score 0, no signs of damage observed on the liver; Score 1, mild or minor lesions confined to under 15% of the liver surface; Score 2, moderate to severe damage involving 30–50% of the liver surface; and Score 3, extensive necrosis of more than 50% of the liver surface. Following scoring, flukes were recovered from the liver. To determine the viability of the *F. hepatica* eggs, they were obtained using the protocols described by Gayo et al. [38]. Briefly, the gallbladders were separated from the livers, and the bile was decanted into a conical vessel. The deposit containing the *F. hepatica* eggs was extensively washed with water. The eggs were then incubated in the dark at 25 °C for 15 days, and egg hatching was induced by exposure to light. Viability was evaluated by estimating the percentage of eggs that hatched into miracidia [39].

### 2.4. ELISA

#### 2.4.1. Total IgG

Anti-*Fh*LAP1 or anti-*Fh*LAP2 total IgG were determined for each serum sample by ELISA, as described in Maggioli et al. [17]. Microplates (Greiner Bio-One, Darmstadt, Germany) were coated with *Fh*LAP1 (2 μg/mL) or *Fh*LAP2 (2 μg/mL) in PBS and incubated overnight at 4 °C. Then, plates were washed three times with PBS containing 0.05% Tween20 (PBS-T) and blocked with PBS-T and 1% BSA overnight at 4 °C. Sera collected from groups *Fh*LAP1, *Fh*LAP1/*Fh*LAP2, IMX, and *Fh*LAP1/Adj50 were diluted in PBS-T and 0.5% BSA and incubated for 1 h at 37 °C. After washing with PBS-T, plates were incubated with HRP-conjugated anti-ovine IgG (Sigma-Aldrich) for 1 h at 37 °C. Following washes, the substrate solution (3,3′,5,5′-Tetramethylbenzidine (TMB) (Sigma-Aldrich) and H_2_O_2_ were added, and the reaction was stopped by adding 50 μL/well of HCl 1 N.

A standard curve was built using a pool of sera from immunized groups, and the antibody concentration of each serum sample was obtained in terms of arbitrary units (AU/mL) by interpolating the optical density readings and multiplying them by the dilution factor.

#### 2.4.2. IgG1 and IgG2 Subclasses

To determine specific IgG subclasses, microplates (Greiner Bio-One, Darmstadt, Germany) were coated with *Fh*LAP1 (2 μg/mL) or *Fh*LAP2 (2 μg/mL) in PBS incubated overnight at 4 °C. Then, plates were washed and blocked as described above.

All sera from groups *Fh*LAP1, *Fh*LAP1/*Fh*LAP2, and IMX were diluted 1:100, and sera *Fh*LAP1/Adj50 were diluted 1:500 in PBS-T and 0.5% BSA. Plates were incubated with anti-ovine IgG1 (1:1000; clone McM1; Pentlands Immunologics, Penicuik, UK) or anti-ovine IgG2 (1:500, clone McM3; Pentlands Immunologics) for 1 h at 37 °C. After washing with PBS-T, plates were incubated with HRP-conjugated anti-mouse IgG (Sigma-Aldrich, St. Louis, MO, USA) for 1 h at 37 °C. Following washes, the substrate TMB and H_2_O_2_ were added, and the reaction was stopped by adding 50 μL/well of HCl 1 N.

IgG subclass titers were expressed in OD 450 nm because they were much lower than those for total IgG levels.

### 2.5. Stimulation and Isolation of Peripheral Blood Leukocytes (PBL)

To study the immune response elicited by our formulations in sheep, changes in cytokine gene expression were assessed in blood samples stimulated in vitro with *Fh*LAP1 or *Fh*LAP1/*Fh*LAP2 antigens, collected in weeks 6 (pre-challenge) and 8 (post-challenge) (Figure 1B). Four animals per group were used to collect blood in a tube containing 1% EDTA. Whole blood was stimulated for 6 h at 37 °C in 5% CO_2_ with 20 µg/mL of *Fh*LAP1 (*Fh*LAP1 group) or 20 µg/mL total *Fh*LAP1/*Fh*LAP2 (*Fh*LAP1/*Fh*LAP2 group), while the control group (IMX group) was stimulated with 20 µg/mL total of *Fh*LAP1 and *Fh*LAP1/*Fh*LAP2 proteins. As a positive control, 1 µg/mL of concanavalin A (Con A, Sigma Aldrich) was used for each blood sample. Then, blood was incubated with red blood cell lysis buffer [40]. The obtained PBLs were thoroughly washed with PBS. Finally, the PBL pellets were conserved in TRIzol reagent at −80 °C.

### 2.6. Gene Expression Analysis

Total RNA from the pre- and post-challenge PBLs obtained above was extracted using the TRIzol protocol (Invitrogen^TM^) according to the manufacturer’s instructions. RNA concentration was measured using a NanoDrop ND-2000 spectrophotometer (NanoDrop Technologies, Wilmington, DE, USA). Only RNA samples with an A280/A260 ratio in the range of 1.8–2.0 and an A260/A230 ratio in the range of 2.0–2.2 were used for cDNA synthesis. Total RNA (1 μg) was treated with 0.4 U of DNase I (Invitrogen, Carlsbad, CA, USA) to remove residual DNA and then reverse transcribed using the SensiFAST cDNA Synthesis Kit (BIO-65053, Meridian Bioscience, Cincinnati, OH, USA), following the manufacturer’s instructions.

Following cDNA synthesis, quantitative PCR (qPCR) was conducted using QuantiTect^®^ SYBR^®^ Green PCR Kit (Qiagen, Hilden, Germany) in an ABI 7900 HT (Applied Biosystems, Foster City, CA, USA) thermocycler. Glyceraldehyde 3-phosphate dehydrogenase (GAPDH) was used as the reference gene, as it has been previously validated by [41] and is widely used in whole blood samples [42,43,44,45]. Prior to relative quantification, we confirmed that its expression remained stable across the different experimental conditions by comparing the Ct value for GAPDH among the groups and times. No significant differences were observed, confirming its suitability as a reference gene.

The primer pairs for IL1β, IL2, IL4, IL5, IL10, IL17, IFNγ, FoxP3, TGFβ, and TNFα mRNA were designed by our group and produced amplicons of the predicted size are showed in Table 1. Cycle program was as follows: initial incubation of 15 min at 95 °C; followed by 40 cycles of 15 s at 95 °C and 1 min at 60 °C with data acquisition; and finally, a melt curve with a ramp from 60 to 95 °C at 1 °C/s. Melt curve analysis was performed to identify and exclude reactions with alternative amplicons.

The relative mRNA amount in each sample was calculated using the 2^−ΔΔCt^ method, as previously described by Livak and Schmittgen [46], where ΔCt = Ct gene of interest—Ct GAPDH. For each cytokine, the mRNA levels in the *Fh*LAP1 and *Fh*LAP1/*Fh*LAP2 groups were expressed relative to the average of the control group (IMX group) stimulated with *Fh*LAP1 or *Fh*LAP1/*Fh*LAP2 proteins, respectively, at pre- and post-challenge (calibration condition).

### 2.7. Exploratory Analysis of Correlations Pre- and Post-Challenge

To analyze the relationships between the changes in cytokine gene expression (IFNγ, TNFα, IL1β, TGFβ, IL10, FoxP3, IL17, and IL2) and the worms recovered (WR) at pre- and post-challenge points within the *Fh*LAP1 or *Fh*LAP1/*Fh*LAP2 groups, the Pearson correlation coefficient was used [47].

### 2.8. Statistical Analysis

Statistical analyses were performed using GraphPad Prism (version 8.00) and PAST (version 4.1) [48]. A *p*-value of <0.05 was considered statistically significant for all analyses.

For the ELISA data, all results were presented as the mean ± standard error (SE). The Kruskal–Wallis test was employed to compare differences between groups for non-parametric data. When significant differences were detected (*p* < 0.05), pairwise comparisons were carried out using Dunn’s post hoc test.

For the cytokine gene expression data, statistical analysis was conducted at each time point (pre- and post-challenge) within each group (*Fh*LAP1 or *Fh*LAP1/*Fh*LAP2). Most data were log-transformed to achieve a more normal distribution. The Shapiro–Wilk test was applied to assess the normality of distributions. For within-group comparisons over time, the paired Student’s t-test [49] was used for parametric data after verifying the assumptions of normality [49] and homogeneity of variances [50]. If these assumptions were not met, a non-parametric paired Wilcoxon test was used [47] (Table 2).

The statistical relationship among variables, specifically worm recovery (WR) and expression of various cytokine genes, was investigated. A Pearson’s correlation test was used to analyze the data after it had been log-transformed to meet the assumption of a normal distribution. *p*-values of 0.05 or lower were considered statistically significant.

## 3. Results

### 3.1. Obtaining Recombinant FhLAP1, FhLAP2, and IMX 

Recombinant *Fh*LAP1 was purified by nickel-chelate affinity chromatography and resulted in a protein of high yield (22.4 mg/L). However, for *Fh*LAP2, the protein refolding procedure resulted in a substantial loss of the protein probable by precipitation. Nevertheless, this procedure improved protein recovery compared to the one described by Checa et al. [20], yielding 1 mg of recombinant *Fh*LAP2/liter of cell culture.

Both recombinant LAPs analyzed by SDS-PAGE showed a high purity level (>90%). The identity of each recombinant protein was confirmed by MALDI-TOF MS/MS. In addition, we obtained the IMX nanoparticles (40 nm) formulated with *Q. brasiliensis* purified saponin fraction by the dialysis method and visualized by HR-TEM (Figure 1A).

### 3.2. Vaccination Efficacy Based on Worm Recovery and Egg Viability

**Figure 2:**
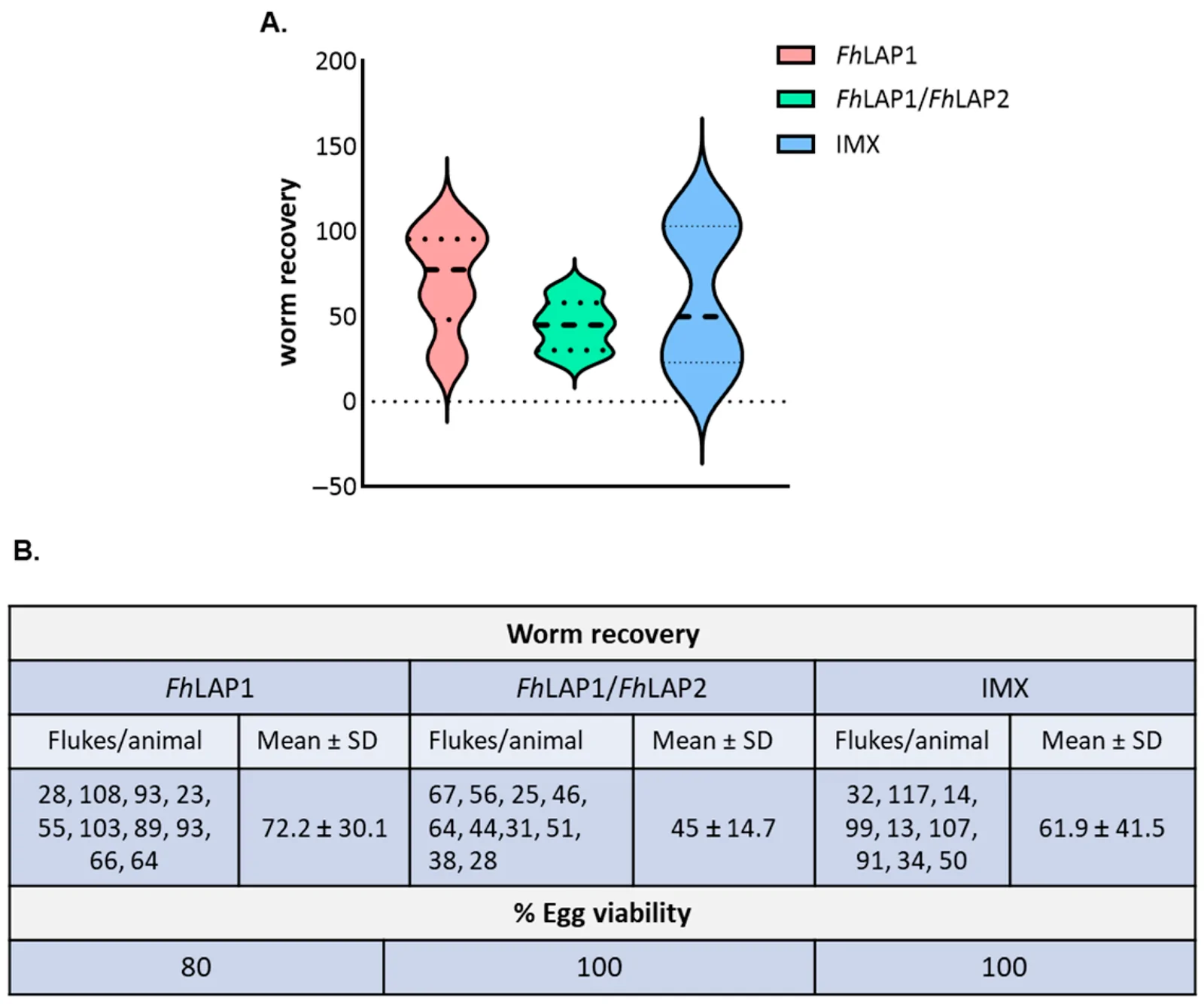
(**A**) Analysis of liver flukes and egg viability at necropsy within *Fh*LAP1, *Fh*LAP1/*Fh*LAP2, and IMX groups. Violin plots illustrate worm recovery variability. Median (striped line), minimum, and maximum (dotted line). (**B**) The data is shown in the table as the mean value ± SD (*n* = 10) and was analyzed by Kruskal Wallis test followed by Dunns’ test. No significant difference was detected.

We carried out the total adult fluke count for each group in the trial (Figure 1B). The fluke burdens were 27.8% lower in the *Fh*LAP1/*Fh*LAP2 group compared to the control IMX vaccinated group (Figure 2B). There is no significant difference compared to the control group IMX. In addition, *Fh*LAP1 and IMX groups showed greater variability compared with the *Fh*LAP1/*Fh*LAP2 group (Figure 2A). Furthermore, the *Fh*LAP1 group displayed a reduction in egg viability (20%) compared to the *Fh*LAP1/*Fh*LAP2 and IMX groups (Figure 2B).

### 3.3. Humoral Response Induced by Vaccination with FhLAP1, FhLAP2, and IMX

**Figure 3:**
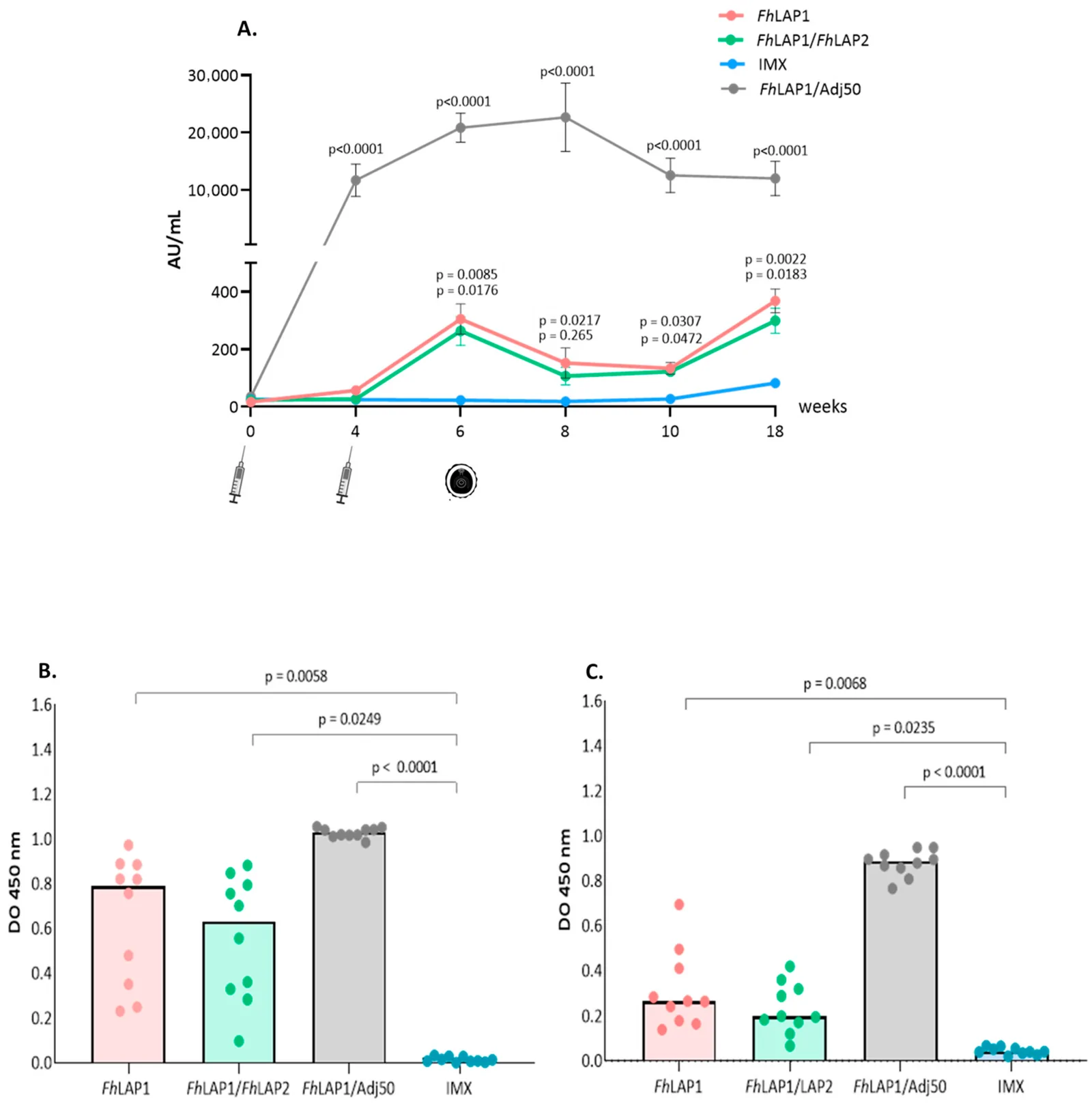
*Fh*LAP1-specific IgG antibodies detection by ELISA. (**A**) Total IgG antibodies were measured in sheep sera (weeks 0, 4, 6, 8, 10, and 18). (**B**) IgG1 and (**C**) IgG2 subclass antibodies were measured in sheep sera at week 6 post-immunization. Serum samples were collected from the sheep every two weeks; syringe icon = immunizations and metacercaria icon = infection. Results have been expressed as the mean value ± SE (*n* = 10). The data were analyzed by Kruskal–Wallis test followed by Dunns’ test.

To assess the humoral immune response induced by the formulations, serum samples collected from sheep were tested for the *Fh*LAP1 or *Fh*LAP2 specific total IgG and the isotypes IgG1 and IgG2 by ELISA. Figure 3 shows the reactivity of sheep sera towards the *Fh*LAP1 in the immunized groups (*Fh*LAP1, *Fh*LAP1/*Fh*LAP2, and *Fh*LAP1/Adj50). As expected, specific total IgG levels were higher in the serum of sheep vaccinated than in the control IMX group.

A significant antibody response was elicited in the *Fh*LAP1 and *Fh*LAP1/*Fh*LAP2 groups two weeks after the second immunization. Then, a decrease in the absorbance of these groups was observed during the 4 weeks after the challenge. In week 10, antibody levels increased, possibly due to liver fluke infection. Nevertheless, the *Fh*LAP1/Adj50 formulation (non-infected group) showed the highest total IgG levels compared to the other three groups (>60-fold). Moreover, this high titer was maintained as high for a more extended period (Figure 3A). Furthermore, significant differences can already be observed with a single dose by week 4.

Levels of IgG1 and IgG2 reacting to the *Fh*LAP1 in sheep from all four groups were measured at week 6 (Figure 3B,C). IgG1 and IgG2 levels were significantly higher in the immunized groups compared with the IMX group. Also, we observed that *Fh*LAP1/Adj50 showed the highest IgG1 and IgG2 levels.

**Figure 4:**
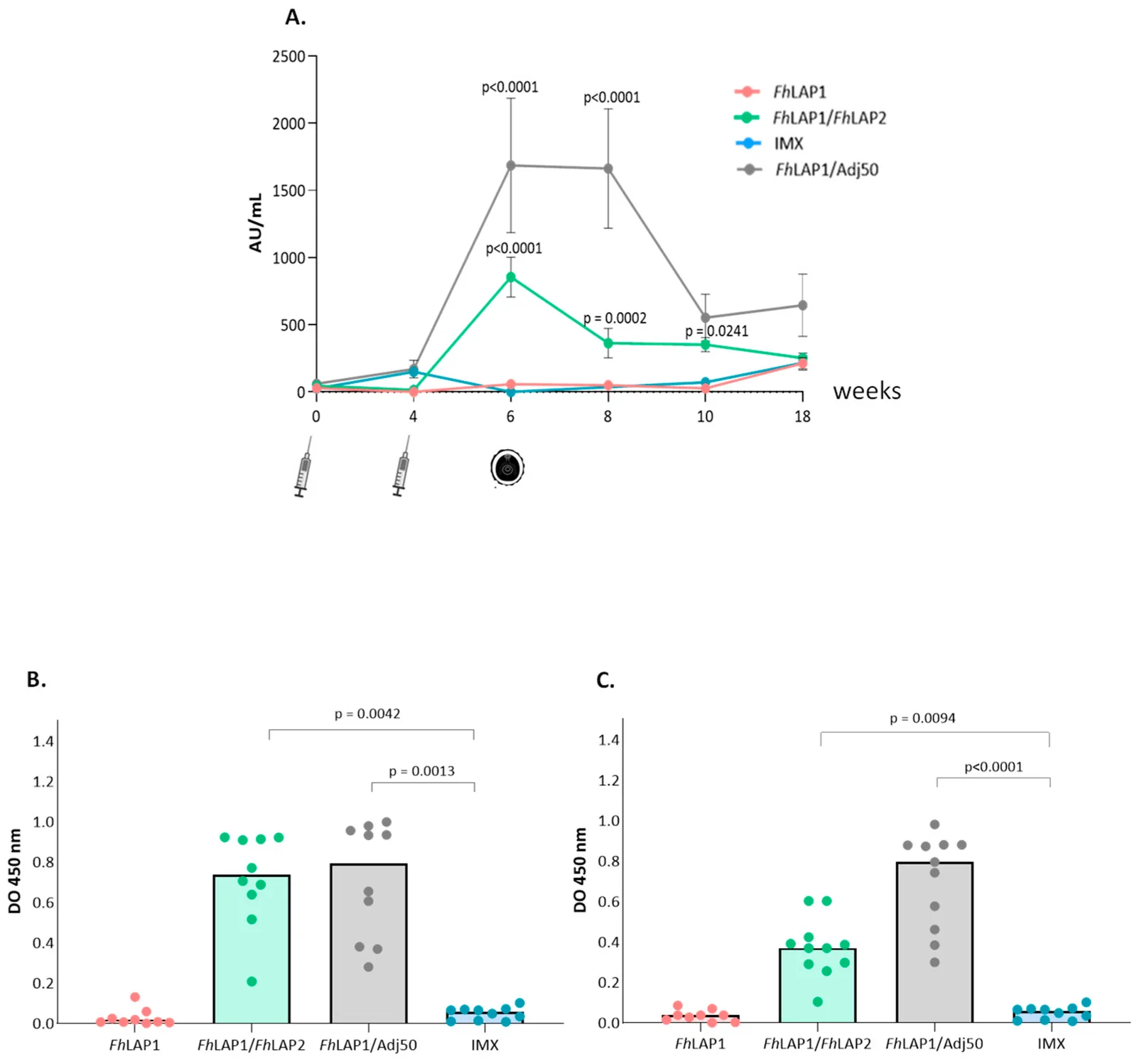
*Fh*LAP2-specific IgG antibodies detection by ELISA. (**A**) Total IgG antibodies were measured in sheep sera (weeks 0, 4, 6, 8, 10, and 18). (**B**) IgG1 and (**C**) IgG2 subclass antibodies were measured in sheep sera at week 6 post-immunization. Serum samples were collected from the sheep every two weeks (syringe) for immunizations and (metacercaria) infection. Results have been expressed as the mean value ± SE (*n* = 10). The data were analyzed by Kruskal–Wallis test followed by Dunns’ test.

The reactivity of sera towards *Fh*LAP2 in the immunized groups shows that the specific total IgG levels were higher in the serum of *Fh*LAP1/FhLAP2 and *Fh*LAP1/Adj50 groups than in the *Fh*LAP1 and IMX groups (Figure 4). A significant antibody response was elicited in the *Fh*LAP1/*Fh*LAP2 and *Fh*LAP1/Adj50 groups two weeks after the second immunization. A decrease in the absorbance of the *Fh*LAP1/*Fh*LAP2 group was observed during the two weeks after the challenge, then antibody levels remained until the end of the trial.

On week 6, IgG1 and IgG2 levels against *Fh*LAP2 were significantly higher in the *Fh*LAP1/*Fh*LAP2 and *Fh*LAP1/Adj50 groups compared with the *Fh*LAP1 and IMX groups (Figure 4B,C).

For the *Fh*LAP1/Adj50 group, we observed the highest total IgG and isotype IgG2 levels compared to the other three groups (Figure 4). This reactivity may be due to the cross-reaction with specific antibodies against the *Fh*LAP1 present in the *Fh*LAP1/Adj50 group; however, this was not observed in the *Fh*LAP1 group.

### 3.4. Gene Expression Levels Induced by Vaccination with FhLAP1 or FhLAP1/FhLAP2

To evaluate the immune response induced by our formulations in sheep, cytokine gene expression changes were assessed in in vitro-stimulated blood samples using *Fh*LAP1 or *Fh*LAP1/*Fh*LAP2 antigens at pre- and post-challenge time points.

**Figure 5:**
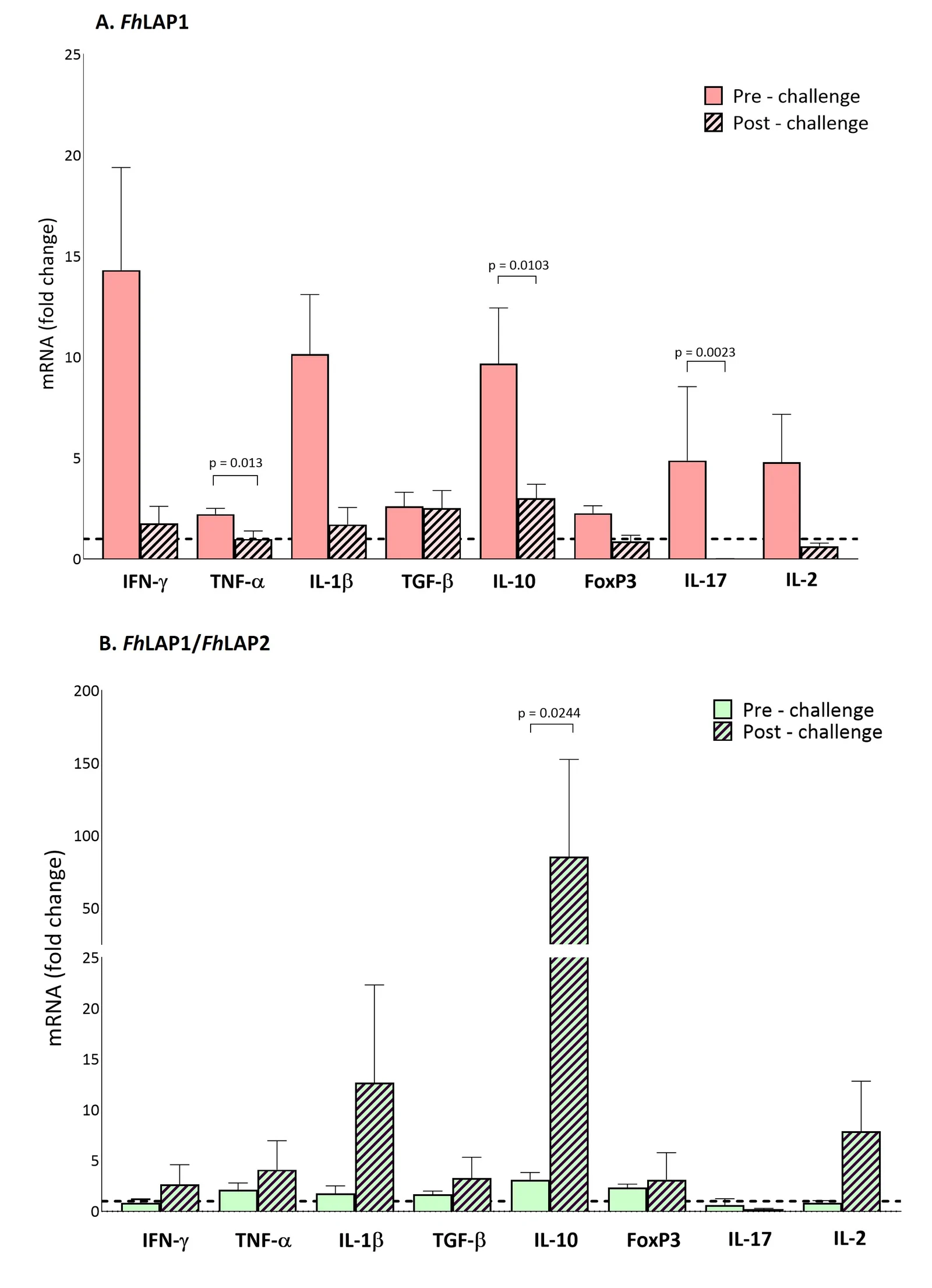
Gene expression levels in PBLs of cytokines (INFγ, TNFα, IL1β, TGFβ, IL10, FoxP3, IL17, and IL2) induced by the (**A**) *Fh*LAP1 and (**B**) *Fh*LAP1/*Fh*LAP2 formulations pre- and post-challenge. Gene expression was analyzed using ΔΔCT [46], normalized with reference gene (GAPDH) and relative to IMX group. Each bar represents the mean ± SEM (*n* = 4). Statistical analysis was performed on log-transformed data using either a parametric paired t-test or a non-parametric paired Wilcoxon test (Table 2). The dotted line indicates a log2 fold-change threshold of 1.0 relative to the IMX group.

In the *Fh*LAP1 group, we detected a significant increase in the pro-inflammatory and regulatory cytokine profile (TNFα, IL10, and IL17) before the challenge. Additionally, we observed an upward trend in IFNγ, IL1β, and IL2 expression at the pre-challenge. After the challenge, the expression levels of cytokines decreased (Figure 5A and Table 2).

In contrast, the *Fh*LAP1/*Fh*LAP2 vaccinated group exhibited a different cytokine profile at both the pre- and post-challenge time points compared to the *Fh*LAP1 group. Before the challenge, no changes in cytokine expression levels were detected relative to the control IMX group. However, after the challenge, a significant shift toward both regulatory (IL10) and pro-inflammatory (TNFα, IL1β, and IL2) responses was observed (Figure 5B and Table 2).

Notably, we were unable to measure Th2-associated cytokines (IL4, IL5) in any experimental group.

### 3.5. Correlation Between Immunological Parameters and Recovery Worm

We then performed a correlation analysis to investigate whether WR might be associated with the immunological parameters analyzed in this study. To this end, pre- and post-challenge cytokine expression levels were assessed within the *Fh*LAP1 and *Fh*LAP1/*Fh*LAP2 groups

**Figure 6:**
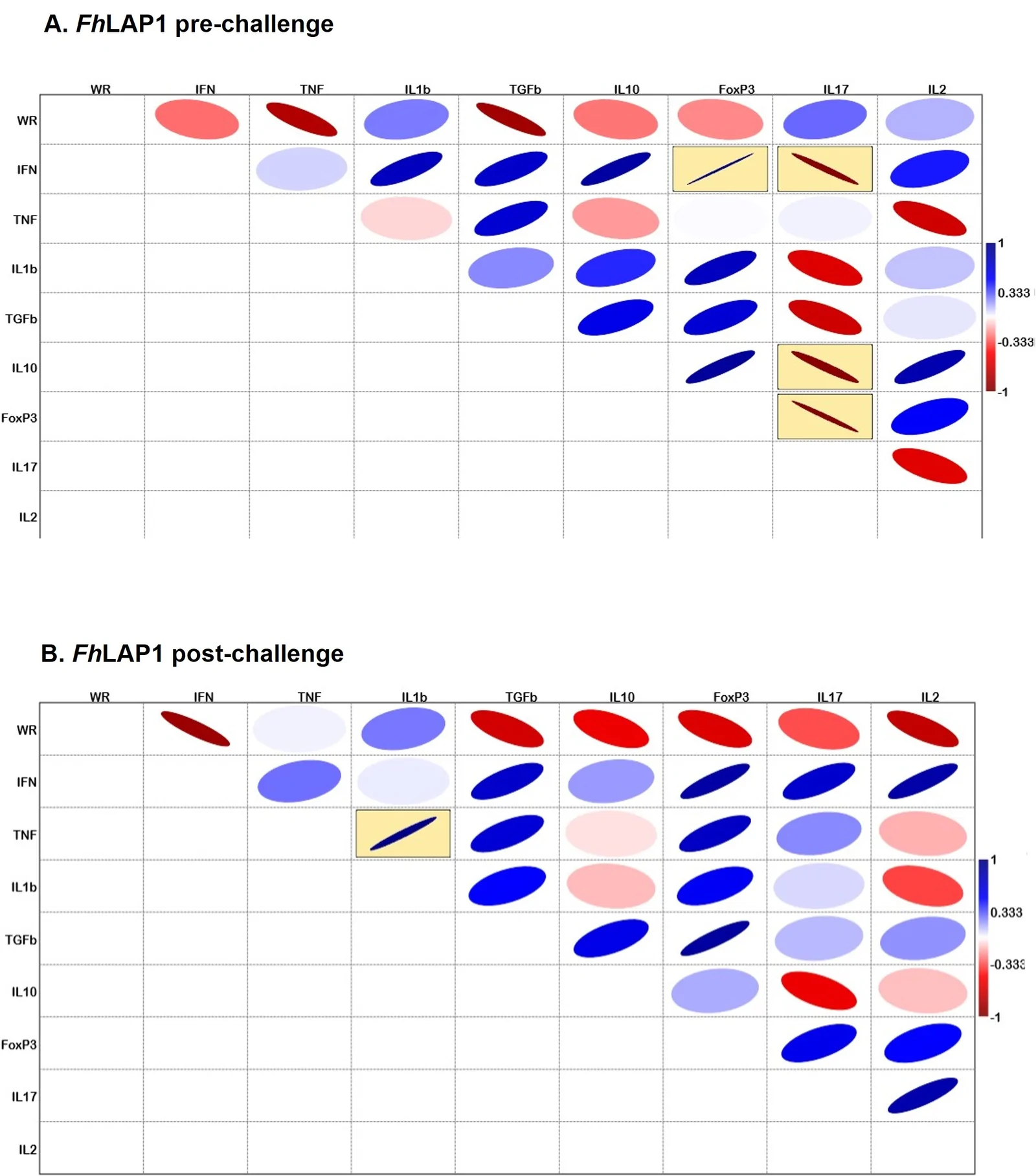
Correlation matrix using Pearson’s correlation coefficient between worm recovery (WR) and gene expression levels of cytokines (IFNγ, TNFα, IL1β, TGFβ, IL10, FoxP3, IL17, and IL2) within the FhLAP1 group. (**A**) Pre-challenge and (**B**) post-challenge points. Blue line indicates positive correlation, and red line indicates negative correlation. The line thickness and color intensity reflect the magnitude of the correlation. Yellow boxes indicate a statistically significant correlation (*p* < 0.05).

**Figure 7:**
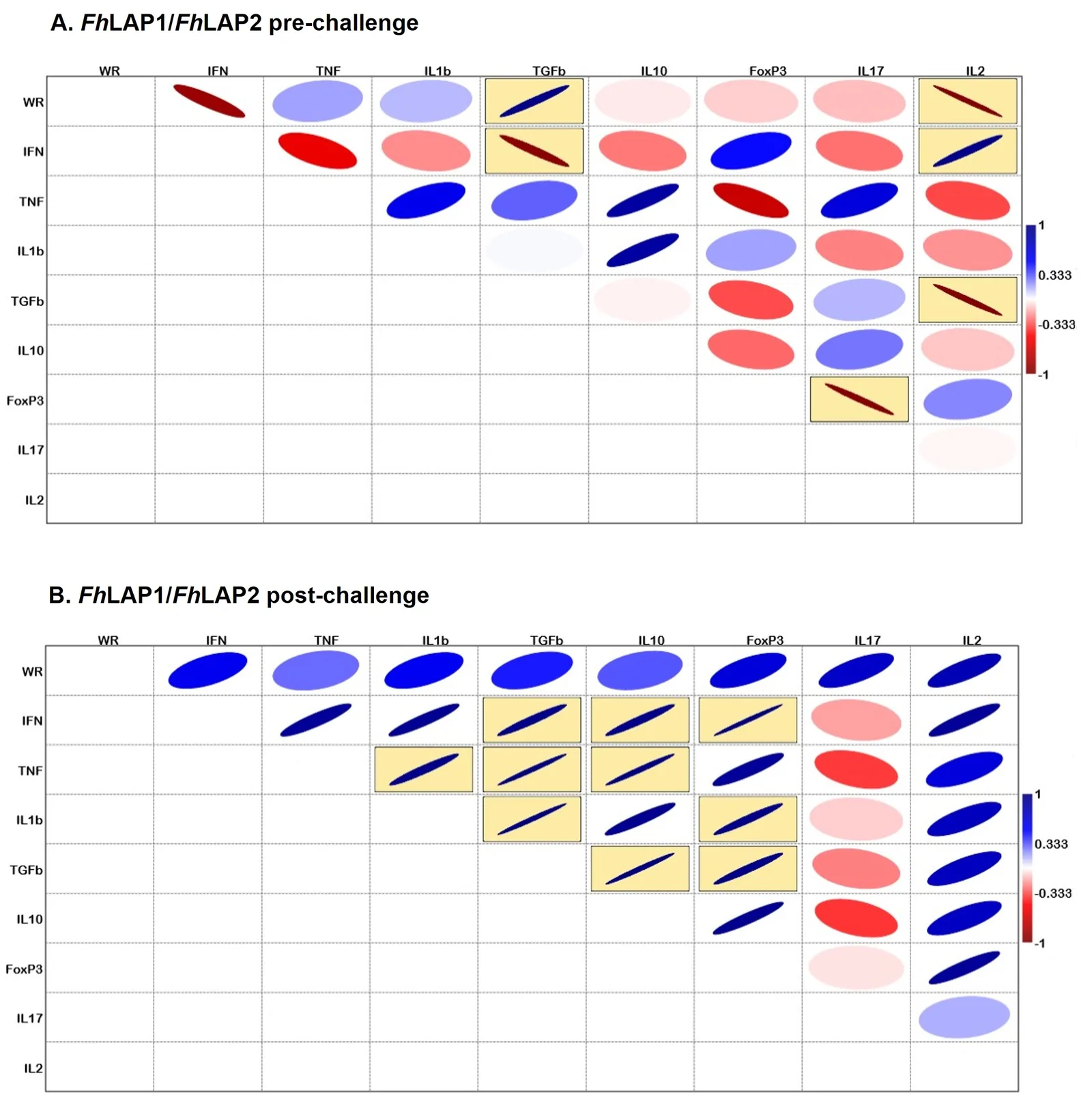
Correlation matrix using Pearson’s correlation coefficient between worm recovery (WR) and gene expression levels of cytokines (IFNγ, TNFα, IL1β, TGFβ, IL10, FoxP3, IL17, and IL2) within the *Fh*LAP1/*Fh*LAP2 group. (**A**) Pre-challenge and (**B**) post-challenge points. Blue line indicates positive correlation, and red line indicates negative correlation. The line thickness and color intensity reflect the magnitude of the correlation. Yellow boxes indicate a statistically significant correlation (*p* < 0.05).

Figure 6 and Figure 7 show correlation matrices based on Pearson’s correlation coefficient, generated from an exploratory analysis. For the *Fh*LAP1 group, we observed a significant positive correlation between FOXP3 and IFNγ and significant negative correlations between IL17 with FOXP3, IFNγ, and IL10 expression levels at the pre-challenge point (Figure 6A). After the challenge, a shift in the correlation profile was observed, with a significant positive correlation between the TNFα and IL1β (Figure 6B), indicating the potential modulation of the immune response during infection.

In the *Fh*LAP1/*Fh*LAP2 group, the pre-challenge analysis revealed a cytokine expression profile distinct from that observed in the *Fh*LAP1 group. IFNγ expression levels showed a positive correlation with both IL2 and TGFβ. Additionally, negative correlations were observed between TGFβ and IL2, as well as between FOXP3 and IL17 (Figure 7A). WR was positively correlated with TGFβ and negatively correlated with IL2.

After the challenge, the expression profile changed, with significant positive correlations observed among several cytokines: IL1β with TNFα; TGFβ with IFNγ, TNFα, and IL1β; IL10 with IFNγ, TNFα, and TGFβ; and FOXP3 with IFNγ, IL1β, and TGFβ. No correlation was found between the WR and changes in cytokine expression (Figure 7B).

## 4. Discussion

Fasciolosis remains a public health and economical problem in many countries, and the identification of vaccine candidates with antiparasitic effects is a challenge in this multicellular parasite. Given the key roles that proteases such as LAPs play in the biological processes of helminths, including digestion, invasion, and migration through the host’s tissues [12,19,51,52], we expected that LAPs-formulated vaccines can reduce the parasite burden. Previous data showed LAPs as the main candidates for vaccine development against fasciolosis, specifically the use of recombinant *Fh*LAP1 combined with different adjuvants, including *Fh*LAP1/Adj50, which has shown relevant protection results in male sheep [17].

Previous studies using recombinant *Fh*LAP1 resulted in fluke burden reductions of 29% and 89%, depending on the vaccine candidate and/or adjuvant used, with highly diverse results [17,20,53,54,55]. Regarding helminths, the use of a recombinant protein cocktail vaccine in ruminants may be more effective than vaccines based on individual antigens [56,57]. We therefore postulate *Fh*LAP2 as an antigen to be used together with *Fh*LAP1 in a bivalent vaccine formulation. *Fh*LAP2 has shown a higher expression in metacercariae and NEJ than *Fh*LAP1 [18,19], and the immunization of mice with *Fh*LAP2/FIA resulted in significant protection against *F. hepatica* infection [20].

Until the present work, these vaccines’ effectiveness in female sheep have not been evaluated. In the female sheep vaccination trial, the mean number of flukes recovered in the IMX group was 61.9 ± 41.5 (range 13–117) and 45 ± 14.7 (range 25–67) in the *Fh*LAP1/*Fh*LAP2 group. Statistical tests showed no significant differences between vaccinated and non-vaccinated female animals, likely due to the high individual variability in the IMX groups (Figure 2). It is noteworthy that two sheep in the IMX group presented low parasite burdens of 13 and 14 flukes. Previous studies have suggested that individual differences in immune responsiveness contribute to the variability of fluke burdens following experimental infection [58,59]. Although evidence on sex-related differences in immune responses remains limited, a vaccination study against fasciolosis in rats suggested potential sex-specific differences, where males exhibited more protective responses than females [60]. In our studies, it is also possible that the variability observed may reflect underlying differences in the type of immune response between the sexes in sheep. There are some reports showing a higher prevalence and intensity of helminth infections in males compared to females, evidencing sex-related differences in the immune response [61,62]. The potential implications of these studies warrant further investigation.

In this work, *Fh*LAP1/Adj50 was used as a reference for IMX-based formulations. These nanoparticles based on *Q. brasiliensis* saponins have been tested in mice with viral antigens polarizing toward a strong Th1 profile [21]. Herein, two fluke antigens, *Fh*LAP1 and *Fh*LAP1/*Fh*LAP2, formulated with IMX, were able to induce a specific total IgG antibody response compared with the IMX control group in ruminants. However, the IgG levels were not as high as those generated by *Fh*LAP1 formulated with Adj50. In accordance with this, other reports using QuilA as an adjuvant in ruminants [58,63,64,65] also showed that using 1 mg/mL QuilA achieved higher levels of specific antibodies compared to those induced by other adjuvants. QuilA injections drive the transition to Th1-type cells, leading to IFN-γ secretion and triggering IgG2 production by B cells. QuilA strongly skews immune responses towards the Th1 type [65], a response aimed to be stimulated to induce protection. In this work, twenty times less saponin was used to prepare the IMX. Although more experimental studies are required, we suggest that it may be necessary to increase the IMX concentration to achieve more robust humoral and inflammatory responses and to sustain them during the infection. The *Fh*LAP1/Adj50 formulation was effective at inducing a strong specific IgG antibody response composed of both IgG1 and IgG2 subtypes, indicating a mixed Th1/Th2 response in female sheep. This result could be in line with evidence associating high antibody levels with protection against *F. hepatica* in ruminants [57,63,66,67]. Previous data showed that male sheep immunized with *Fh*LAP1/Adj50 had a significant fluke burden reduction and induced a mixed Th1/Th2 immune response [17]. So, it would be reasonable to assume that this formulation would also reduce the worm burden in female sheep.

Several studies have demonstrated that oil-based adjuvants promote high and durable antibody titers but induce intolerable reactogenicity, such as abscess and cyst formation at the injection site [68,69], which negatively impacts the meat and hides of livestock [70]. An alternative is the use of saponins extracted from the leaves of *Q. brasiliensis* without the need to cut down the tree, formulated as nanoparticles (IMX). In addition, they exhibit greater biodegradability compared with oil-based adjuvants. The present work demonstrated the induction of mixed Th1/Th2 responses using *Fh*LAPs in female sheep that potentially protect against fasciolosis. Additional challenge trials using a high number of females are needed to confirm the antiparasitic effect of the *Fh*LAPs/IMX formulation.

When the immune profile through the gene expression of Th1/Th2/Th17/Treg cytokines was analyzed, the *Fh*LAP1/*Fh*LAP2 group showed a trend toward a pro-inflammatory and regulatory profile compared with the *Fh*LAP1 group after the challenge. We observed that infection with *F. hepatica* might change the cellular immune profile initially generated by the formulations, which could be beneficial for worm establishment [71,72]. Protective immunity requires a dominant Th1 response [29]. Chronic infection promotes an immunoregulatory environment with the suppression of parasite-specific Th1/Th2 responses and increased IL-10 [73,74,75]. In this study, cytokines associated with a Th2-type response (IL-4, IL-5, or IL-13) were not evaluated, and therefore, their impact could not be assessed. However, we observed that *Fh*LAP1/IMX formulations stimulated a Th1-type response profile before the challenge, but this profile was not sustained after infection. With the *Fh*LAP1/*Fh*LAP2 formulation, the post-infection profile appeared more appropriate; however, a robust IFN-γ expression, indicative of protective immunity, was not achieved. The presence of IL-10 was observed, suggesting a suppressive Th1/Th2 response more consistent with that developed during a chronic infection [66].

To date, it remains unclear which specific immune mechanisms need to be induced by vaccination. Taking everything into account, we suggest that a strong humoral immune response, coupled with the selection of an appropriate adjuvant, is required for the specific inhibition of these enzymes, which may compromise the survival of the parasite by impeding vital processes. Strategic adjuvant selection is essential to enhance vaccine immunogenicity and stimulate the production of immunologically active molecules, thereby inducing stronger and longer-lasting protective immunity [76,77,78]. In this sense, it would be necessary to generate a robust humoral immune response in addition to stimulating greater production of IFN-γ, indicating a Th1 profile immune stimulus. Our data suggests that future trials should consider increasing the IMX concentration or using an oil-based adjuvant such as Adj50 to generate a more robust humoral immune response in addition to stimulating a Th1 profile immune response.

## 5. Conclusions

In this study, we evaluated *Fh*LAP-based formulations using IMX as the adjuvant that was capable of inducing a regulatory Th1/Th2 immune environment in ruminants. No significant differences in the worm burden were observed, probably due to high variability in the IMX control group inherent to characteristics of this natural host such as sexor breed. These findings provide valuable insights into the design of vaccination trials in ruminants. They will guide future studies aimed at refining candidate selection, optimizing formulations, evaluating novel adjuvants and delivery systems, and ensuring statistically robust group sizes for *F. hepatica* vaccination. Additional studies are warranted to assess this strategy in ruminants.

## Figures and Tables

**Table 1:** Description and sequences of the primers designed to quantify specific ovine genes using qPCR.

Primer	Seq 5′-3′	Amplicon Size (bp)
FoxP3	F: CCCTTTCACCTATGCCACCC R: ATCTCGTTGAGTGTCCGCTG	75
IL1β	F: CTGGTGCTGGATAGCCCAT R: GTACGAAGCTCATGCAGAACA	91
IL2	F: AGAGAGATCAAGGATTCAATGGAC R. TTACTGTCGCATCATCATATTCAC	100
IL4	F: CGCTGAACATCCTCACATCG R: CTCAGTTGCGTTCTTTGGGG	86
IL5	F: TCCTCAGCATACAAATCACCAAC R: CAGCATCCCCTTGTGCAGT	89
IL10	F: CCCTGCGAAAACAAGAGCAAG R: CACTCATGGCTTTGTAGACACC	88
IL17	F: GGCGTCTATGAGAACTGCCT R: ATGACCCCTGCCTTCACAAG	75
INFγ	F: ACTGGAGGACTTCAAAAGGCT R: ATTGATGGCTTTGCGCTGGAT	70
TNFα	F: GCACTTCGGGGTAATCGGC R: GCTTGAGAAGATGACCTGAGTGT	99
GAPDH [41]	F: GGCGTGAACCACGAGAAGTATAA R: CCCTCCACGATGCCAAAGT	120

**Table 2:** Statistical analysis summary for evaluating changes in cytokine gene expression levels. Student test (t), Wilcoxon test (W). Significant * *p* < 0.05 and ** *p*-values = 0.023; ns: not significant.

Group	Cytokine	Statistic Test
*Fh*LAP1	IL-10	t = 5.782 *
	TNF-α	t = 3.755 *
	IL-17	t = 9.802 **
	IL-1β	W = −10 ns
	TGF-β	t = 0.6095 ns
	INF-γ	t = 1.474 ns
	FoxP3	t = 2.916 ns
	IL-2	W = −10 ns
*Fh*LAP1/*Fh*LAP2	IL-10	t = 4.214 *
	TNF-α	t = 0.3767 ns
	IL-17	t = 1.198 ns
	IL-1β	W = 10 ns
	TGF-β	t = 0.5337 ns
	INF-γ	t = 0.3863 ns
	FoxP3	W = −6 ns
	IL-2	W = 2 ns

## Data Availability

The original contributions presented in this study are included in the article. Further inquiries can be directed to the corresponding author.
